# Sex differences in risk factors for myocardial infarction: cohort study of UK Biobank participants

**DOI:** 10.1136/bmj.k4247

**Published:** 2018-11-07

**Authors:** Elizabeth R C Millett, Sanne A E Peters, Mark Woodward

**Affiliations:** 1 The George Institute for Global Health, University of Oxford, Oxford OX1 2BQ, UK; 2 Julius Center for Health Sciences and Primary Care, University Medical Center Utrecht, Utrecht, Netherlands; 3 The George Institute for Global Health, University of New South Wales, Newtown, NSW, Australia; 4 Department of Epidemiology, Johns Hopkins University, Baltimore, MD, USA

## Abstract

**Objectives:**

To investigate sex differences in risk factors for incident myocardial infarction (MI) and whether they vary with age.

**Design:**

Prospective population based study.

**Setting:**

UK Biobank.

**Participants:**

471 998 participants (56% women; mean age 56.2) with no history of cardiovascular disease.

**Main outcome measure:**

Incident (fatal and non-fatal) MI.

**Results:**

5081 participants (1463 (28.8%) of whom were women) had MI over seven years’ mean follow-up, resulting in an incidence per 10 000 person years of 7.76 (95% confidence interval 7.37 to 8.16) for women and 24.35 (23.57 to 25.16) for men. Higher blood pressure indices, smoking intensity, body mass index, and the presence of diabetes were associated with an increased risk of MI in men and women, but associations were attenuated with age. In women, systolic blood pressure and hypertension, smoking status and intensity, and diabetes were associated with higher hazard ratios for MI compared with men: ratio of hazard ratios 1.09 (95% confidence interval 1.02 to 1.16) for systolic blood pressure, 1.55 (1.32 to 1.83) for current smoking, 2.91 (1.56 to 5.45) for type 1 diabetes, and 1.47 (1.16 to 1.87) for type 2 diabetes. There was no evidence that any of these ratios of hazard ratios decreased with age (P>0.2). With the exception of type 1 diabetes, the incidence of MI was higher in men than in women for all risk factors.

**Conclusions:**

Although the incidence of MI was higher in men than in women, several risk factors were more strongly associated with MI in women compared with men. Sex specific associations between risk factors and MI declined with age, but, where it occurred, the higher relative risk in women remained. As the population ages and the prevalence of lifestyle associated risk factors increase, the incidence of MI in women will likely become more similar to that in men.

## Introduction

Coronary heart disease (CHD) has been the leading cause of mortality worldwide for over 25 years,^1^ ^2^ and was estimated to be the cause of 17% of deaths globally in 2016.^3^ Death rates from CHD are considerably lower in women than in men at younger ages, but often converge with increasing age. Male-to-female coronary mortality rate ratios are typically around 4 to 5 in middle age (30-64) and 2 thereafter (65-89).^4^ In the INTERHEART case-control study, women had their first myocardial infarction (MI) on average nine years later than men.^5^ In addition to later presentation, men and women can have different symptoms, treatments, and outcomes of MI, some of which may be because of the effects and prevalence of risk factors. Several large scale meta-analyses have compared the sex specific associations between risk factors and CHD. Key findings from these analyses are that, compared with men, women had a higher ratio of relative risk of CHD: 44% higher if they had diabetes^6^ and 25% higher if they were current smokers.^7^ However, these meta-analyses included studies performed over an extensive time scale, with heterogeneous study populations, and with varying sets of adjustment for potential confounders. Importantly, the analyses were unable to reliably explore whether the identified sex differences in relative risk were consistent with age, or to compare the sex differences for different risk factors on an equal basis. In addition, the analyses could not make comparisons on the absolute scale.

To obtain comparably adjusted results, overall and within age groups, we used the UK Biobank to investigate the sex differences in risk factors for MI and how these may vary with age.

## Methods

The UK Biobank is a large prospective study of 502 628 participants recruited between 2006 and 2010.^8^ Participants aged between 40 and 69 were invited to attend one of 22 centres for a baseline assessment, where informed consent was obtained, a touchscreen questionnaire was completed, a face-to-face interview was conducted, and a range of physical measurements were taken. Participants gave details of their medical history, regular use of any drugs, and their lifestyle factors, such as smoking status. Baseline data are linked to hospital admissions data (hospital episode statistics admitted patient care activity (HES, England), the general/acute inpatient and day case dataset (SMR01, Scotland) and the patient episode database for Wales (PEDW)) and Office for National Statistics (ONS) mortality records, which enable long term follow-up of participants and their health outcomes.

Participants who subsequently withdrew from the study (n=64) and those with a history of cardiovascular disease (self reported or hospital admission diagnosis of MI, angina, or stroke before the date of the baseline assessment, n=30 566) were excluded from the current analyses.

### Measurement of risk factors

We investigated six risk factors: blood pressure, smoking status, diabetes mellitus, body mass index, atrial fibrillation, and socioeconomic status. For blood pressure, the mean of two sitting systolic and diastolic blood pressure measurements, taken at baseline using the Omron HEM-7015IT digital blood pressure monitor (Omron Healthcare), was calculated. Blood pressure was categorised using American Heart Association (AHA) 2017 guidelines (normal: systolic blood pressure <120 mm Hg and diastolic blood pressure <80 mm Hg; elevated: systolic blood pressure 120-129 mm Hg and diastolic blood pressure <80 mm Hg; stage 1 hypertension: systolic blood pressure 130-139 mm Hg or diastolic blood pressure 80-89 mm Hg; and stage 2 hypertension: systolic blood pressure ≥140 mm Hg or diastolic blood pressure ≥90 mm Hg).^9^ We further categorised AHA stages into eight groups according to reported use of antihypertensive drugs. Smoking status was self reported and included the daily cigarette consumption of current smokers. Self reported diabetes was categorised as type 1 if participants were aged less than 30 when the disease was diagnosed and they were using insulin, and type 2 otherwise. Participants with type 2 diabetes were further classified according to self reported treatment. Body mass index was calculated by dividing a participant’s weight in kilograms, measured using the Tanita BC-418 MA body composition analyser (Tanita Corporation of America), by the square of his or her standing height in metres, measured using a Seca 202 height measure (SECA, Germany). Overweight was defined as a body mass index of at least 25 kg/m^2^ and less than 30 kg/m^2^; obesity was defined as a body mass index of 30 kg/m^2^ or higher. A history of atrial fibrillation was self reported. Socioeconomic status was determined using the postcode based Townsend deprivation index and categorised into thirds using national cut-off points (high: <–2.08; middle ≥–2.08 to <1.40; low ≥1.40).

### Outcomes

The study endpoint was the incidence of fatal or non-fatal MI, defined using an algorithm developed by UK Biobank.^10^ Linking with relevant hospital admissions data (HES, SMR01, and PEDW) and ONS enabled UK Biobank to identify the date of the first known MI after the date of baseline assessment by using codes I21, I22, I23, I24.1 or I25.2 from the 10th edition of the International Classification of Diseases. Follow-up for all participants started at inclusion in the UK Biobank and ended on 31 March 2016, or when the first fatal or non-fatal MI occurred.

### Statistical analyses

Baseline characteristics for women and men are presented as number (percentage) for categorical variables, mean (standard deviation) for approximately symmetrical continuous variables, and median (interquartile range) for asymmetrical continuous variables. The incidence of MI was estimated separately for women and men. Cox proportional hazard models were used to estimate hazard ratios and 95% confidence intervals for MI comparing women with men, adjusted for age, systolic blood pressure, body mass index, smoking status, and diabetes. These analyses were further stratified by age in five year groups.

To estimate the hazard ratios for MI for each risk factor, we used Cox regression models, with interaction terms between each variable and sex. All models were adjusted for age and other variables specified a priori, which varied across risk factors. In addition to age, we adjusted systolic blood pressure, diabetes, and socioeconomic status for each other as well as for smoking status, body mass index, use of lipid lowering drugs, and antihypertensive drugs. Atrial fibrillation was similarly adjusted for these eight variables. Diastolic blood pressure and AHA hypertension stages were adjusted for the same variables as systolic blood pressure, except when AHA hypertension stage was further categorised by use of antihypertensive drugs, in which case adjustment for antihypertensive drugs was clearly inappropriate. The models for smoking variables included socioeconomic status, and the models for body mass index included smoking status and socioeconomic status. We decided not to adjust body mass index for systolic blood pressure because systolic blood pressure is thought to be a mediating factor and our goal was to examine the independent effects of sex on risk factor associations with MI.

The interaction term of each risk factor with sex was used to obtain the women-to-men relative hazard ratio for each risk factor. This ratio should be interpreted as a measure of interaction (in a statistical sense) or effect modification (in a clinical sense). We used a likelihood ratio test to check for deviation from the linear trend for the ratio of hazard ratios for categories of daily cigarette consumption.

Missing data can be a source of bias. We determined the percentage of missing data for each variable used in this study. We ran a sensitivity analysis for any variable for which more than 5% of the data were missing by performing 20 iterations of a multiple imputation using chained equations and including sex interactions in the imputation model.^11^ When less than 5% of data for a risk factor or potential confounder of interest was missing, participants with missing data were not included in the relevant model.

To investigate whether sex differences in risk factors differed by age group (<50, 50-59, and ≥60), we added a three way interaction between sex, age group, and the risk factor of interest to the models. Age group was subsequently included as a linear term to produce Wald test P values for trend among women and men and in the interaction for the ratio of hazard ratios. Because the UK Biobank cohort is comparatively socially advantaged (and healthy) compared with the UK population in general, we investigated whether there was evidence of heterogeneity in the effects of risk factors according to socioeconomic status. Therefore, we ran additional subgroup analyses for participants at or above, and below the national median Townsend deprivation index score (−0.56).

We also looked at sex differences on the absolute scale, which are less likely to be relevant than relative risks for application in other populations, but should be considered when making clinical decisions. We evaluated sex differences on the absolute scale as unadjusted and adjusted rates per 10 000 person years by sex and their women-to-men difference of differences, estimated using Poisson regression models. Adjustments were the same as those used in the Cox models.

Analyses were performed using Stata version 14.2.

### Patient and public involvement

No patients were involved in setting the research question or the outcome measures, and they were not involved in developing plans for the design or implementation of the study. No patients were asked to advise on interpretation or writing up of results. We have no plans to disseminate the results of the research to study participants or the relevant patient community.

## Results

Table 1 presents the baseline characteristics of 471 998 participants with no history of cardiovascular disease included in these analyses. The proportion of women was 56% and the mean age at study baseline was 56 (standard deviation 8) in both sexes. At baseline, a lower percentage of women than men had diabetes or atrial fibrillation, or were taking lipid lowering or blood pressure lowering drugs. Overall, women had slightly lower blood pressure and were less likely to have ever smoked than men.

**Table 1:** Characteristics of women and men without a history of cardiovascular disease who participated in the UK Biobank. Values are numbers (percentages) unless stated otherwise

Characteristics	Women (n=263 323)	Men (n=208 675)
**Incident myocardial infarction during follow-up, No (% of total MIs)**	**1 463 (28.8)**	**3 618 (71.2)**
Mean (SD) age (years)	56.2 (8.0)	56.3 (8.2)
Ethnicity:		
White	248 045 (94.2)	195 990 (93.9)
Other*	14 050 (5.3)	11 302 (5.4)
Blood pressure (mm Hg):		
Mean (SD) systolic	135.2 (19.2)	141.1 (17.4)
Mean (SD) diastolic	80.8 (10.0)	84.5 (9.9)
AHA hypertension categories:		
Normal	53 770 (20.5)	17 316 (8.3)
Elevated	34 192 (13.0)	23 256 (11.2)
Stage 1 hypertension	115 930 (44.1)	103 317 (49.6)
Stage 2 hypertension	58 707 (22.4)	64 278 (30.9)
Smoking status:		
Never smoker	157 200 (60.0)	104 720 (50.5)
Former smoker	81 611 (31.2)	76 922 (37.1)
Current smoker	23 083 (8.8)	25 776 (12.4)
Mean (SD) cigarettes smoked daily (current smokers)	14 (7.0)	17 (9.0)
Smoking intensity (average No of cigarettes smoked daily):		
1-9	4 241 (18.4)	2 571 (10.0)
10-19	7 953 (34.5)	6 262 (24.3)
≥20	5 200 (22.5)	6 961 (27.0)
Not reported	5 689 (24.6)	9 982 (38.7)
Mean (SD) years smoked (current smokers)	36.0 (9.3)	36.9 (9.9)
Body mass index (kg/m^2^):		
Mean (SD) body mass index	27.0 (5.2)	27.7 (4.2)
Overweight (≥25, <30)	96 136 (36.7)	102 942 (49.7)
Obese (≥30)	60 343 (23.0)	50 364 (24.3)
Socioeconomic status:		
Median (interquartile range) Townsend deprivation index score	−2.17 (−3.65-0.42)	−2.17 (−3.67-0.54)
Townsend deprivation thirds:		
Low	49 396 (18.8)	41 000 (19.7)
Middle	78 929 (30.0)	60 777 (29.2)
High	134 681 (51.2)	106 625 (51.2)
Diabetes:		
Type 1†	500 (0.2)	531 (0.3)
Type 2	8 631 (3.3)	11 852 (5.7)
Type 2 treatment:		
No drugs reported	3 497 (40.5)	4 275 (36.1)
Oral drugs only	4 000 (46.3)	6 018 (50.8)
Insulin only	492 (5.7)	670 (5.7)
Oral drugs and insulin	642 (7.4)	889 (7.5)
Mean (SD) type 2 diagnosis (years)	6.7 (7.2)	7.2 (8.1)
Atrial fibrillation:		
History of atrial fibrillation	985 (0.4)	2 006 (1.0)
Drug use:		
Antihypertensive drugs	32 340 (12.3)	32 563 (15.6)
Lipid lowering drugs	23 067 (8.8)	29 544 (14.2)

AHA=American Heart Association.

* Includes Asian or Asian British, black or black British, Caribbean, African, any other black background, Chinese, other ethnic group, white and black Caribbean, white and black African, white and Asian, any other mixed background, Indian, Pakistani, Bangladeshi, any other Asian background.

† Defined as diagnosis at age <30 and receiving insulin treatment.

After a mean follow-up of seven years, 5081 first MI events were recorded (1463 (28.8%) in women) (table 1). Models adjusted for multiple variables showed that the rate of MI in women was less than half that in men (hazard ratio 0.37; 95% confidence interval 0.35 to 0.40). The lower risk of MI in women compared with men was apparent across all age groups, but attenuated slightly with increasing age, from 0.27 (0.18 to 0.41) among those aged less than 45, to 0.45 (0.40 to 0.50) among those aged 65 and over (supplementary table 1).

### Blood pressure

**Fig 1:**
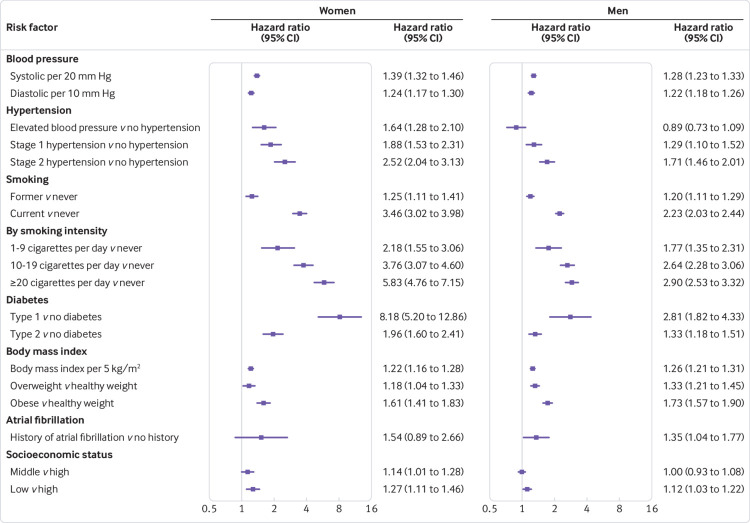
Adjusted hazard ratios for association between risk factors and incident myocardial infarction by sex. Horizontal lines indicate corresponding 95% confidence intervals around hazard ratios. All models were adjusted for age. Additionally, systolic blood pressure, diabetes, and socioeconomic status were adjusted for each other as well as smoking status, body mass index, lipid lowering drugs, and antihypertensive drugs. Atrial fibrillation was similarly adjusted for these eight variables. Diastolic blood pressure and American Heart Association hypertension stages were adjusted for the same variables as systolic blood pressure. Models for smoking variables included socioeconomic status, and models for body mass index contained smoking status and socioeconomic status

**Fig 2:**
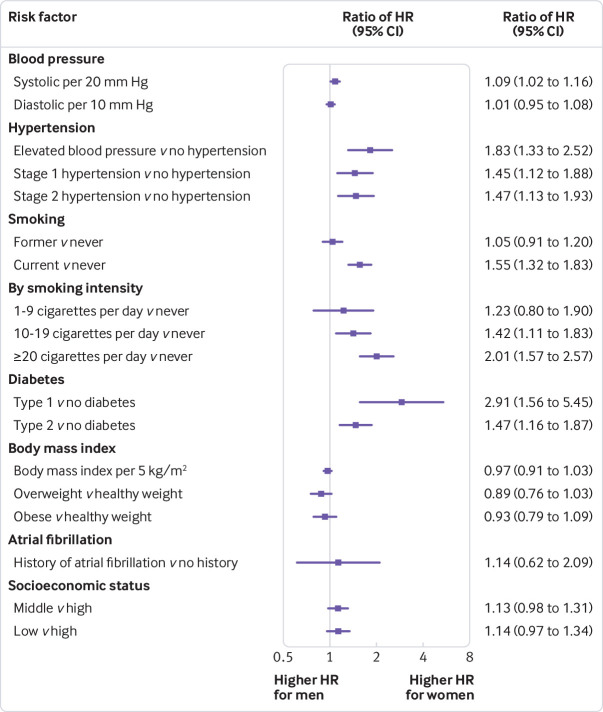
Adjusted women-to-men ratios of hazard ratios for association between risk factors and incident myocardial infarction. Horizontal lines indicate corresponding 95% confidence intervals around ratio of hazard ratios. All models were adjusted for age. Additionally, systolic blood pressure, diabetes, and socioeconomic status were adjusted for each other as well as smoking status, body mass index, lipid lowering drugs, and antihypertensive drugs. Atrial fibrillation was similarly adjusted for these eight variables. Diastolic blood pressure and American Heart Association hypertension stages were adjusted for the same variables as systolic blood pressure. Models for smoking variables included socioeconomic status, and models for body mass index contained smoking status and socioeconomic status

Rising values of all blood pressure indices were associated with a higher risk of MI in both sexes after adjusting for confounding variables (fig 1). Compared with normal blood pressure, the risk of MI increased with AHA hypertension stage in both sexes and was consistently higher among women than men. The women-to-men ratio of hazard ratios for elevated blood pressure was 1.83 (95% confidence interval 1.33 to 2.52), whereas for stage 1 and stage 2 hypertension the ratios were both about 1.5 (fig 2). We also considered the effects of higher blood pressure on MI according to antihypertensive drug use. The hazard ratios for MI were higher for participants taking drugs than for those not taking drugs for each AHA stage and in both sexes (supplementary table 2). This increase was more pronounced in women. For example, comparing participants with elevated blood pressure taking antihypertensive drugs with those with normal blood pressure not taking antihypertensive drugs, the hazard ratio was 3.65 (2.44 to 5.44) in women and 1.75 (1.26 to 2.44) in men (ratio of hazard ratios 2.08, 95% confidence interval 1.24 to 3.50) (supplementary table 2).

### Smoking status

Compared with never smoking, current and former smoking were each associated with an increased risk of MI in both sexes, but with larger hazard ratios in women than men. For current smokers, the hazard ratios were 3.46 (95% confidence interval 3.02 to 3.98) in women and 2.23 (2.03 to 2.44) in men (fig 1) (ratio of hazard ratios 1.55, 95% confidence interval 1.32 to 1.83; fig 2). Greater smoking intensity was associated with a higher risk of MI in both sexes, but especially among women. The women to men ratio of hazard ratios increased with cigarette consumption (with evidence of non-linearity, P=0.006; fig 2).

### Type 1 and 2 diabetes

Compared with people without diabetes, women with type 1 diabetes had more than eight times the risk of MI (hazard ratio 8.18, 95% confidence interval 5.20 to 12.86; fig 1), almost three times the hazard ratio in men (2.81, 1.82 to 4.33; fig 1; ratio of hazard ratios 2.91, 1.56 to 5.45; fig 2). Type 2 diabetes was associated with an increased risk of MI in both sexes; the hazard ratio was 1.96 (1.60 to 2.41) in women and 1.33 (1.18 to 1.51) in men (fig 1); the ratio of hazard ratios was 1.47 (1.16 to 1.87; fig 2). When analysing the type of treatment given to people with type 2 diabetes, there was still an excess risk in women compared with men (supplementary table 3). Hazard ratios for both sexes and the women-to-men ratio were greater when insulin use was reported.

### Body mass index

The risk of MI rose with increasing body mass index, and with being overweight or obese (compared with normal weight) for both sexes (fig 1). However, there was no evidence of any difference by sex (ratio of hazard ratios 0.97 (95% confidence interval 0.91 to 1.03) for every 5 kg/m^2^ additional body mass index, 0.89 (0.76 to 1.03) for overweight, and 0.93 (0.79 to 1.09) for obese; fig 2).

### Atrial fibrillation

The hazard ratio for MI associated with atrial fibrillation was 1.54 (95% confidence interval 0.89 to 2.66) in women and 1.35 (1.04 to 1.77) in men (fig 1); there was no evidence of a sex difference (ratio of hazard ratios 1.14, 95% confidence interval 0.62 to 2.09; fig 2).

### Socioeconomic status

Compared with high socioeconomic status, women with a low socioeconomic status had a hazard ratio for MI of 1.27 (95% confidence interval 1.11 to 1.46); the equivalent hazard ratio in men was 1.12 (1.03 to 1.22) (fig 1). For each unit increase in the Townsend deprivation index score (moving from higher to lower socioeconomic status), women had 3% (hazard ratio 1.03, 1.02 to 1.05) higher risk of MI compared with a 2% (1.02, 1.01 to 1.03) higher risk in men. There was some evidence that this risk was slightly higher among women than men (ratio of hazard ratios 1.02, 1.00 to 1.04).

### Confounding and modification by age

The results from the age adjusted analyses were generally similar to the multiple adjusted analyses (supplementary table 4). The sex specific hazard ratios for stage 1 and 2 hypertension, current smokers, diabetes, and socioeconomic status were slightly higher than those that were further adjusted, as were the ratios of hazard ratios for current smokers, diabetes, atrial fibrillation, and the lowest third category of socioeconomic status.

**Fig 3:**
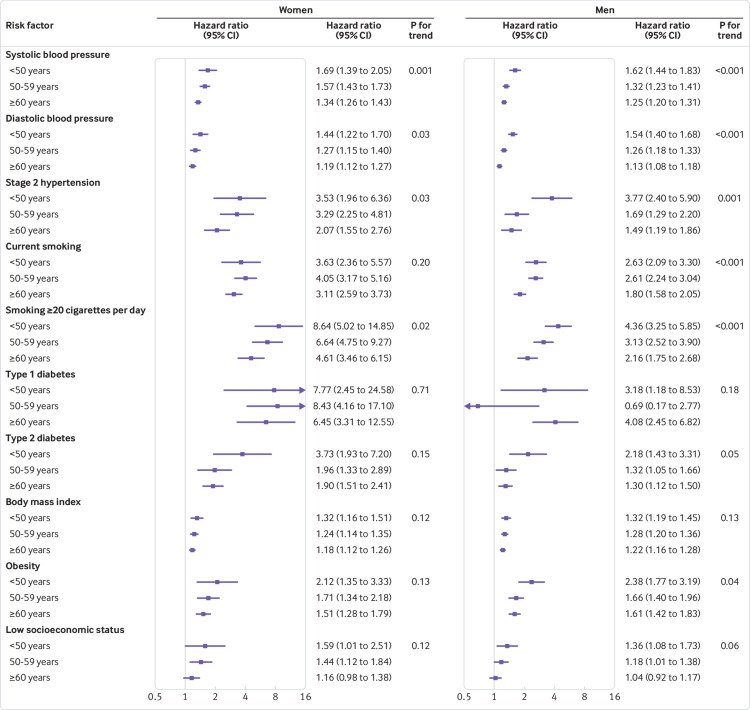
Adjusted hazard ratios for association between risk factors and incident myocardial infarction by age group and sex. Horizontal lines indicate corresponding 95% confidence intervals around hazard ratios. Systolic blood pressure is given per 20 mm Hg and diastolic blood pressure per 10 mm Hg. Participants with stage 2 hypertension were compared with participants with normal blood pressure; current smokers were compared with never smokers; participants with diabetes were compared with those without diabetes; obesity was compared with body mass index less than 25 kg/m^2^; and for socioeconomic status the lowest third was compared with the highest third. Models for systolic blood pressure, diabetes and socioeconomic status were adjusted for each other as well as smoking status, body mass index, lipid lowering drugs, and antihypertensive drugs. Diastolic blood pressure and American Heart Association hypertension stages were adjusted for the same variables as systolic blood pressure. Models for smoking variables included socioeconomic status, and models for body mass index contained smoking status and socioeconomic status

**Fig 4:**
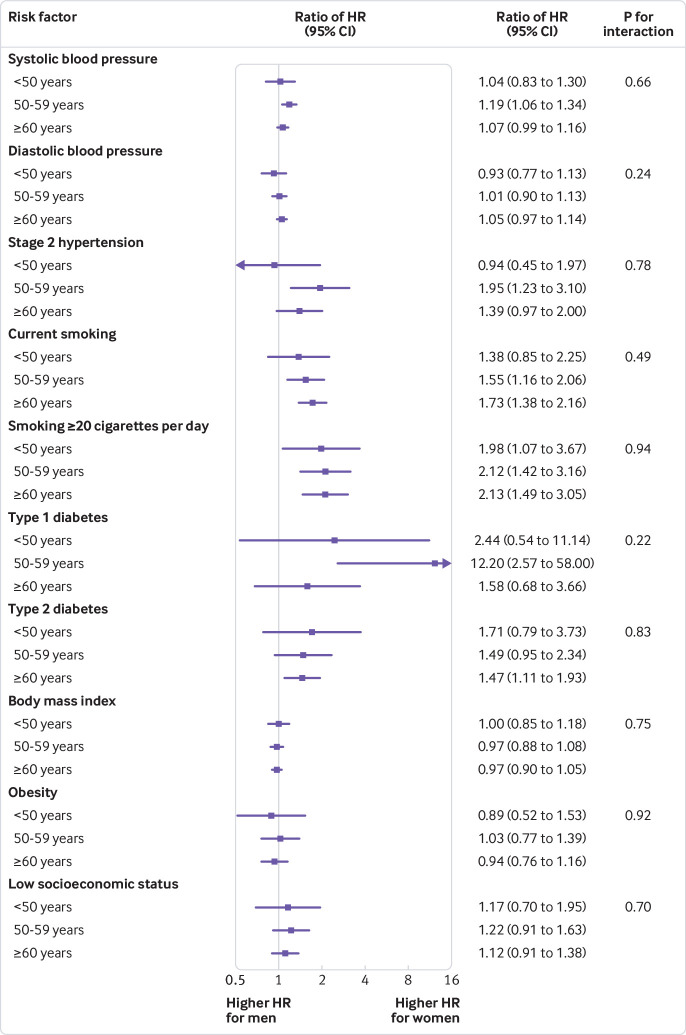
Adjusted women-to-men ratios of hazard ratios for association between risk factors and incident myocardial infarction by age group. Horizontal lines indicate corresponding 95% confidence intervals around ratio of hazard ratios. Systolic blood pressure is given per 20 mm Hg and diastolic blood pressure per 10 mm Hg. Participants with stage 2 hypertension were compared with participants with normal blood pressure; current smokers were compared with never smokers; participants with diabetes were compared with those without diabetes; obesity was compared with body mass index less than 25 kg/m^2^; and for socioeconomic status the lowest third was compared with the highest third. Models for systolic blood pressure, diabetes, and socioeconomic status were adjusted for each other as well as smoking status, body mass index, lipid lowering drugs, and antihypertensive drugs. Diastolic blood pressure and American Heart Association hypertension stages were adjusted for the same variables as systolic blood pressure. Models for smoking variables included socioeconomic status, and models for body mass index contained smoking status and socioeconomic status

In both sexes, the hazard ratios for systolic blood pressure, diastolic blood pressure, hypertension stage 2, and high smoking intensity decreased with increasing age (fig 3). Type 2 diabetes was associated with a higher risk of MI in those aged less than 50 (women: hazard ratio 3.73 (95% confidence interval 1.93 to 7.20); men: 2.18 (1.43 to 3.31); fig 3), while the 50-59 and 60 and older age groups had similarly increased risks of around 90% in women and 30% in men. For none of the risk factors investigated was there evidence that the women-to-men ratio of hazard ratios differed by age group (fig 4 and supplementary table 5).

### Modification by socioeconomic status

The only evidence of heterogeneity by socioeconomic status in the effects of sex on risk factors for MI was for former smoking versus never smoking (P=0.004, supplementary table 6). Among women with low socioeconomic status, former smokers had a 49% increased risk of MI compared with non-smokers. This increase was not present among men (hazard ratio 1.06, 95% confidence interval 0.93 to 1.22; ratio of hazard ratios 1.41, 1.11 to 1.79). There was no evidence of a sex difference in the hazard ratios comparing former with never smokers among those with high socioeconomic status (supplementary table 6).

### Sex comparisons of rates of MI

Finally, we describe sex differences on the absolute scale. Incidence rates of MI per 10 000 person years were 7.76 (95% confidence interval 7.37 to 8.16) in women and 24.35 (23.57 to 25.16) in men. Type 1 diabetes was the only risk factor associated with comparable rates of MI in women and men. For all other risk factors, and for every category of these risk factors, men had higher rates of MI than women (table 2; supplementary table 7). The unadjusted rates of MI were generally similar to the multiple adjusted rates, except among participants with type 1 diabetes in whom rates increased after adjustment, and among those with type 2 diabetes or atrial fibrillation in whom rates decreased after adjustment. In both sexes, multiple adjusted rates were highest among those with type 1 diabetes (women: 64.92/10 000 person years (95% confidence interval 35.88 to 93.96); men: 61.92/10 000 person years (35.17 to 88.67); table 2).

**Table 2:** Multiple adjusted rates of myocardial infarction (per 10 000 person years) by sex, and women-to-men difference of rate differences for each risk factor

Variables	Rates/10 000 person years (95% CI)		Difference of rate differences (95% CI)
	Women (n=263 323)	Men (n=208 675)	
AHA hypertension categories:			
Normal	4.32 (3.49 to 5.15)	16.88 (14.33 to 19.43)	—
Elevated	7.07 (5.92 to 8.21)	15.04 (13.05 to 17.03)	4.59 (1.09 to 8.09)
Stage 1 hypertension	8.23 (7.60 to 8.87)	21.85 (20.79 to 22.91)	−1.05 (−4.00 to 1.90)
Stage 2 hypertension	11.18 (10.19 to 12.18)	28.92 (27.42 to 30.41)	−5.17 (−8.42 to −1.92)
Smoking status:			
Never smoker	6.09 (5.62 to 6.55)	19.84 (18.79 to 20.90)	—
Former smoker	7.63 (6.94 to 8.31)	23.76 (22.52 to 24.99)	−2.37 (−4.20 to −0.55)
Current smoker	21.01 (18.61 to 23.41)	44.06 (40.83 to 47.29)	−9.29 (−13.49 to −5.10)
Smoking intensity (average No of cigarettes smoked daily):			
Never*	5.70 (5.26 to 6.13)	18.99 (17.98 to 19.99)	—
1-9	12.41 (8.29 to 16.53)	33.62 (24.79 to 42.46)	−7.93 (−17.74 to 1.89)
10-19	21.33 (17.37 to 25.28)	49.95 (43.11 to 56.79)	−15.34 (−23.33 to −7.35)
≥20	33.00 (26.82 to 39.17)	54.73 (47.86 to 61.59)	−8.44 (−17.76 to 0.88)
Diabetes:			
No diabetes	7.95 (7.50 to 8.40)	22.06 (21.27 to 22.84)	—
Type 1*	64.92 (35.88 to 93.96)	61.92 (35.17 to 88.67)	17.11 (−22.39 to 56.61)
Type 2	15.63 (12.64 to 18.62)	29.41 (25.92 to 32.91)	0.33 (−4.40 to 5.05)
Body mass index (kg/m^2^):			
Normal (<25)	6.66 (6.04 to 7.28)	17.62 (16.30 to 18.93)	—
Overweight (≥25, <30)	7.82 (7.16 to 8.49)	23.38 (22.28 to 24.48)	−4.59 (−6.53 to −2.65)
Obesity (≥30)	10.69 (9.69 to 11.68)	30.47 (28.66 to 32.29)	−8.83 (−11.36 to −6.30)
Atrial fibrillation:			
None	8.59 (8.13 to 9.04)	22.47 (21.71 to 23.23)	—
History of atrial fibrillation	13.19 (5.97 to 20.40)	30.41 (22.37 to 38.45)	−3.34 (−14.18 to 7.50)
Townsend deprivation thirds:			
High	7.84 (7.21 to 8.46)	22.00 (20.97 to 23.03)	—
Middle	8.93 (8.10 to 9.77)	22.11 (20.73 to 23.50)	0.98 (−1.03 to 2.99)
Low	10.00 (8.92 to 11.08)	24.66 (22.86 to 26.45)	−0.50 (−2.92 to 1.93)

AHA=American Heart Association.

All models were adjusted for age. Additionally, systolic blood pressure, diabetes, and socioeconomic status were adjusted for each other as well as smoking status, body mass index, lipid lowering drugs, and antihypertensive drugs. Atrial fibrillation was similarly adjusted for these eight variables. Diastolic blood pressure and AHA hypertension stages were adjusted for the same variables as systolic blood pressure. The models for smoking variables included socioeconomic status, and the models for body mass index contained smoking status and socioeconomic status.

* Smoking status and smoking intensity models produced slightly different adjusted rates of MI for never smokers. This is because of the effect of adjustment for covariables differing slightly in each model, as they contained different smoking variables, and former smokers and those with missing cigarette consumption were excluded from the smoking intensity model.

* Defined as diagnosis at age <30 and receiving insulin treatment.

## Discussion

This study of 471 998 middle aged UK Biobank participants with no history of cardiovascular disease analysed the sex differences in risk factors for myocardial infarction (MI). Women who smoked more than 20 cigarettes per day had twice the relative risk of MI than equivalent men, and elevated blood pressure was associated with a more than 80% higher relative risk in women. Hypertension stages 1 and 2, smoking 10-19 cigarettes daily, and type 2 diabetes each were 40% more strongly associated with the risk of MI in women than men.

Since the sex specific relative risks attenuated with age in both sexes, our results suggest that cardiovascular risk scores should consider including age interactions for greater predictive accuracy. However, we found no evidence to suggest that the women-to-men comparisons were significantly different across age groups.

### Strengths and limitations of this study

Previous studies of sex differences in MI have been restricted to a single risk factor, pooled data from disparate studies, or were based on hospital populations. In this study we analysed sex differences in MI across a range of risk factors in a general population using standardised methods on the relative and absolute scales. We also examined how age impacts sex differences in MI. The only comparable study examining a similar range of risk factors in men and women and using a standard protocol is INTERHEART. This, however, was a retrospective case-control study of patients admitted to hospital with their first acute MI, and hospital sourced controls. The study design is inferior to the UK Biobank cohort design and is more susceptible to bias.^12^

Our study has limitations. Some variables had missing data but the only variable with more than 5% of data missing was the number of cigarettes smoked daily (supplementary table 8). After multiple imputation, the effects of smoking intensity, however, were not appreciably different from the complete case analysis (supplementary table 9) and we conclude that the primary results are valid. Most UK Biobank participants are white and further work is required to assess the generalisability of the results to other populations. Higher socioeconomic status was over represented in the study population, which could have limited the ability of this study to find sex differences among different socioeconomic groups. Blood samples were taken from all participants but at the time of analysis the lipid profile data were not available, preventing examination of this as a risk factor and adjusting for it as a confounder. Instead we adjusted analyses for participants’ use of lipid lowering drugs when appropriate, but residual confounding by lipid levels is possible. Diagnoses and recording of drug use at baseline relied on self report, which will have resulted in some errors. However, these errors were probably minor and may have been the same in both sexes. UK Biobank participants were aged between 40 and 70 at recruitment, and so sex differences among younger and older populations were not analysed. Whether the additional risk associated with some factors varies across a wider age range would be an interesting topic for future research.

### Comparison with other studies

This study adds to the growing literature on potential sex differences in risk factors for cardiovascular disease. In a meta-analysis of more than 900 000 people, the increased risk of ischaemic heart disease with each 10 mm Hg rise in systolic blood pressure was found to be 13% in women and men.^13^ There was important heterogeneity between studies, which could partly be because of different trends in blood pressure worldwide.^14^ Our findings are similar to those from a recent English study of 1.25 million patients and 11 029 MI events, in which a slightly higher relative risk of MI with increasing systolic blood pressure, but not diastolic blood pressure, was found in women compared with men.^15^ INTERHEART reported higher odds ratios of MI in women with hypertension than in men with hypertension (odds ratio 2.95 (95% confidence interval 2.66 to 3.28) in women and 2.32 (2.16 to 2.48) in men).^5^ We found a higher relative risk of MI in women compared with men across all American Heart Association (AHA) hypertension stages, and this was most pronounced among those with elevated blood pressure. Women may be less likely to receive blood pressure lowering drugs and be less compliant with treatment, resulting in poorer blood pressure control than men.^16^ Women’s longer exposure to the effects of hypertension (including before any treatment) could explain some of the higher relative risk we found in our analyses.

The INTERHEART study found that smoking accounted for around 36% of the population attributable risk of MI worldwide.^12^ Both men and women who were current smokers had around three times the odds of MI compared with never smokers. There was evidence of a sex difference by former smoking status. Women who were former smokers were not found to have increased odds of MI compared with never smokers, whereas men who were former smokers had around 60% increased odds of MI compared with never smokers.^5^ Our previous meta-analysis described a women to men ratio of relative risks of CHD of 1.25 (95% confidence interval 1.12 to 1.39) in current smokers compared with non-smokers,^7^ which is lower than the ratio of hazard ratios of 1.55 in current smokers compared with never smokers found in the current study. In agreement with the current study, this meta-analysis found no evidence of a sex difference when comparing the risk of CHD between former smokers and never smokers.^7^ INTERHEART and the Tromsø study reported an association between rising cigarette consumption and an increasing risk of MI in both sexes.^17^ ^18^ Although the Tromsø study reported no evidence of an interaction between sex and smoking intensity, the effect sizes in both studies were larger among women than men.^17^ ^18^ Our estimate of a higher relative risk in female, than male, current smokers compared with never smokers, and with increasing cigarette consumption, may be explained by differences in the duration of the tobacco epidemic worldwide; in many countries the epidemic among women is relatively recent, leading to an underestimate of women’s excess relative risk in some studies.^19^ ^20^ We have previously described how the smoking habits in UK Biobank men and women have become increasingly similar over time,^21^ providing a more direct women-to-men comparison of the risk of MI associated with smoking.

The sex difference in risk of cardiovascular disease in people with diabetes has been subject to increasing interest over recent years. Pooled analyses of over 800 000 people, including over 26 000 incident CHD events, showed that women with diabetes had a 44% excess relative risk of incident CHD compared with men with diabetes.^6^ INTERHEART and an Italian cohort study reported higher risks of MI in women with diabetes than in men,^5^ ^22^ whereas a meta-analysis and the China Kadoorie Biobank described an excess relative risk of mortality from CHD in women with diabetes compared with men.^23^ ^24^ In contrast, a large English cohort study of 1.9 million patients observed a slightly increased risk of non-fatal MI in women with type 2 diabetes aged less than 60 than in men of a similar age, but no differences among older age groups.^25^ Exclusion of fatal MI may be partly responsible for their results, as MI mortality has been found to be higher in women than in men with diabetes.^26^ Deterioration in cardiovascular risk factor levels among those with and without type 2 diabetes is greater in women than in men; therefore women with diabetes are at a disadvantage compared with men, even before their diagnosis.^27^ Additionally, in the UK women with diabetes are 15% less likely than men with diabetes to meet all recommended care requirements, and might be less likely to achieve target values for treated cardiovascular risk factors.^28^ ^29^ ^30^ The cumulative effects of these disadvantages among women throughout the trajectory of disease could explain some of the excess relative risk.

### Can women “catch up” with men?

An interesting question is under what conditions would rates of MI in men and women be the same? The answer depends on many factors, including the prevalence of the risk factors for MI (see supplementary appendix). In the UK Biobank the rates of MI per 10 000 person years in women with hypertension (AHA stage 1 or 2) and diabetes (type 1 or 2) who were also current smokers was 41.76 (95% confidence interval 25.58 to 68.12) compared with 53.68 (39.95 to 72.13) in men. So even in this extreme group, defined only by risk factors for which relative risks in women exceed those in men, in our study population men still have higher rates of MI. Furthermore, few women (0.2%) and men (0.6%) had this combination of risk factors and their contribution to the overall rate of MI is inevitably small. It would take an enormous increase in the prevalence of all three risk factors for this subgroup to have even a moderate impact on the overall risk of MI in the future.

### Conclusions and policy implications

Although the risk of MI is, on average, about three times higher in men than women, women tend to “catch up” to some extent if they have certain cardiovascular risk factors. Our findings suggest that clinicians should be vigilant when their female patients are elderly, smoke, have diabetes, or have high blood pressure. These findings also highlight the importance of equitable access to guideline based treatments for diabetes and hypertension, and to weight loss and smoking cessation programmes for women and men in middle and older age.

Despite the rate of MI being higher in men than women, hypertension, smoking (especially higher intensity), and type 1 and 2 diabetes confer a greater excess risk of MI in women than in men. This excess risk does not attenuate with age. In addition, a rising prevalence of lifestyle associated risk factors, coupled with the ageing population, is likely to result in women having a more similar overall rate of MI to men in the future, with a major additional burden on society and health resources.

**What is already known on this topic**

The incidence of myocardial infarction (MI) is lower in women than in men at younger ages, but the incidence becomes more similar with increasing age
Meta-analyses have shown sex differences in the association between several risk factors and MI, but the studies included had varying levels of adjustment for confounders and could not examine sex differences by age group

**What this study adds**

Hypertension, smoking, and diabetes were associated with an increased risk of MI in women and men, but with an excess relative risk in women
Although the sex specific associations between these risk factors and MI attenuated with age, the excess relative risk of MI in women did not
Women and men should receive the same access to guideline based treatments for diabetes and hypertension, and to resources to help them lose weight and stop smoking

## Web extra

Extra material supplied by authors

**Supplementary information:** supplementary tables 1-9, and equations

## Notes

Ethical approval: UK Biobank has obtained Research Tissue Bank approval from its governing research ethics committee, as recommended by the National Research Ethics Service. No separate ethical approval was required. The study was conducted in accordance with the principles of the Declaration of Helsinki.

Data sharing: Researchers can apply to use the UK Biobank resource and access the data used. No additional data are available.

Transparency: The manuscript’s guarantor (ERCM) affirms that this manuscript is an honest, accurate, and transparent account of the study being reported; that no important aspects of the study have been omitted; and that any discrepancies from the study as planned (and, if relevant, registered) have been explained.

## References

[1] World Health Organization. Top 10 causes of death worldwide Geneva: WHO; 2017. www.who.int/mediacentre/factsheets/fs310/en/.

[2] Institute for Health Metrics and Evaluation. GBD Compare Data Visualization Seattle, WA: IHME, University of Washington; 2016. https://vizhub.healthdata.org/gbd-compare/.

[3] Naghavi M Abajobir AA Abbafati C GBD 2016 Causes of Death Collaborators Global, regional, and national age-sex specific mortality for 264 causes of death, 1980-2016: a systematic analysis for the Global Burden of Disease Study 2016. Lancet 2017;390:1151-210. 10.1016/S0140-6736(17)32152-9 28919116 PMC5605883

[4] Bots SH Peters SAE Woodward M Sex differences in coronary heart disease and stroke mortality: a global assessment of the effect of ageing between 1980 and 2010. BMJ Global Health 2017:2:e000298 10.1136/bmjgh-2017-000298 PMC5435266 28589033

[5] Anand SS Islam S Rosengren A INTERHEART Investigators Risk factors for myocardial infarction in women and men: insights from the INTERHEART study. Eur Heart J 2008;29:932-40. 10.1093/eurheartj/ehn018 18334475

[6] Peters SAE Huxley RR Woodward M Diabetes as risk factor for incident coronary heart disease in women compared with men: a systematic review and meta-analysis of 64 cohorts including 858,507 individuals and 28,203 coronary events. Diabetologia 2014;57:1542-51. 10.1007/s00125-014-3260-6 24859435

[7] Huxley RR Woodward M Cigarette smoking as a risk factor for coronary heart disease in women compared with men: a systematic review and meta-analysis of prospective cohort studies. Lancet 2011;378:1297-305. 10.1016/S0140-6736(11)60781-2 21839503

[8] Sudlow C Gallacher J Allen N UK biobank: an open access resource for identifying the causes of a wide range of complex diseases of middle and old age. PLoS Med 2015;12:e1001779. 10.1371/journal.pmed.1001779 25826379 PMC4380465

[9] Whelton PK Carey RM Aronow WS ACC/AHA/AAPA/ABC/ACPM/AGS/APhA/ASH/ASPC/NMA/PCNA Guideline for the Prevention, Detection, Evaluation, and Management of High Blood Pressure in Adults. A Report of the American College of Cardiology/American Heart Association Task Force on Clinical Practice Guidelines. Hypertension 2017;71:e13-e115 10.1161/HYP.0000000000000065. 29133356

[10] Schnier C, Sudlow C. Definitions of acute myocardial infarction (MI) and main MI pathological types for UK Biobank phase 1 outcomes adjudication 2017 [14 September 2018]. https://biobank.ctsu.ox.ac.uk/crystal/docs/alg_outcome_mi.pdf.

[11] Woodward M Epidemiology: Study Design and Data Analysis. 3rd ed Chapman and Hall/CRC, 2013 10.1201/b16343.

[12] Yusuf S Hawken S Ôunpuu S INTERHEART Study Investigators Effect of potentially modifiable risk factors associated with myocardial infarction in 52 countries (the INTERHEART study): case-control study. Lancet 2004;364:937-52. 10.1016/S0140-6736(04)17018-9 15364185

[13] Peters SAE Huxley RR Woodward M Comparison of the sex-specific associations between systolic blood pressure and the risk of cardiovascular disease: a systematic review and meta-analysis of 124 cohort studies, including 1.2 million individuals. Stroke 2013;44:2394-401. 10.1161/STROKEAHA.113.001624 23821229

[14] Zhou B Bentham J Di Cesare M Worldwide trends in blood pressure from 1975 to 2015: a pooled analysis of 1479 population-based measurement studies with 19.1 million participants. Lancet 2017;389:37-55. 27863813 10.1016/S0140-6736(16)31919-5 PMC5220163

[15] Rapsomaniki E Timmis A George J Blood pressure and incidence of twelve cardiovascular diseases: lifetime risks, healthy life-years lost, and age-specific associations in 1·25 million people. Lancet 2014;383:1899-911. 10.1016/S0140-6736(14)60685-1 24881994 PMC4042017

[16] Tamargo J Rosano G Walther T Gender differences in the effects of cardiovascular drugs. Eur Heart J Cardiovasc Pharmacother 2017;3:163-82. 10.1093/ehjcvp/pvw042 28329228

[17] Teo KK Ounpuu S Hawken S INTERHEART Study Investigators Tobacco use and risk of myocardial infarction in 52 countries in the INTERHEART study: a case-control study. Lancet 2006;368:647-58. 10.1016/S0140-6736(06)69249-0 16920470

[18] Iversen B Jacobsen BK Løchen M-L Active and passive smoking and the risk of myocardial infarction in 24,968 men and women during 11 year of follow-up: the Tromsø Study. Eur J Epidemiol 2013;28:659-67. 10.1007/s10654-013-9785-z 23443581 PMC3779067

[19] Ng M Freeman MK Fleming TD Smoking prevalence and cigarette consumption in 187 countries, 1980-2012. JAMA 2014;311:183-92. 10.1001/jama.2013.284692 24399557

[20] Hitchman SC Fong GT Gender empowerment and female-to-male smoking prevalence ratios. Bull World Health Organ 2011;89:195-202. 10.2471/BLT.10.079905 21379415 PMC3044247

[21] Peters SAE Huxley RR Woodward M Do smoking habits differ between women and men in contemporary Western populations? Evidence from half a million people in the UK Biobank study. BMJ Open 2014;4:e005663. 10.1136/bmjopen-2014-005663 25550291 PMC4281541

[22] Ballotari P Venturelli F Greci M Giorgi Rossi P Manicardi V Sex Differences in the Effect of Type 2 Diabetes on Major Cardiovascular Diseases: Results from a Population-Based Study in Italy. Int J Endocrinol 2017;2017:6039356. 10.1155/2017/6039356 28316624 PMC5338069

[23] Gnatiuc L Herrington WG Halsey J Prospective Studies Collaboration and Asia Pacific Cohort Studies Collaboration Sex-specific relevance of diabetes to occlusive vascular and other mortality: a collaborative meta-analysis of individual data from 980 793 adults from 68 prospective studies. Lancet Diabetes Endocrinol 2018;6:538-46. 10.1016/S2213-8587(18)30079-2 29752194 PMC6008496

[24] Bragg F Holmes MV Iona A China Kadoorie Biobank Collaborative Group Association between diabetes and cause-specific mortality in rural and urban areas of china. JAMA 2017;317:280-9. 10.1001/jama.2016.19720 28114552 PMC6520233

[25] Shah AD Langenberg C Rapsomaniki E Type 2 diabetes and incidence of cardiovascular diseases: a cohort study in 1·9 million people. Lancet Diabetes Endocrinol 2015;3:105-13. 10.1016/S2213-8587(14)70219-0 25466521 PMC4303913

[26] Roche MM Wang PP Sex differences in all-cause and cardiovascular mortality, hospitalization for individuals with and without diabetes, and patients with diabetes diagnosed early and late. Diabetes Care 2013;36:2582-90. 10.2337/dc12-1272 23564923 PMC3747934

[27] Wannamethee SG Papacosta O Lawlor DA Do women exhibit greater differences in established and novel risk factors between diabetes and non-diabetes than men? The British Regional Heart Study and British Women’s Heart Health Study. Diabetologia 2012;55:80-7. 10.1007/s00125-011-2284-4 21861177

[28] Health and Social Care Information Centre National Diabetes Audit - 2012-2013: Report 1. Care Processes and Treatment Targets, 2014.

[29] Franzini L Ardigò D Cavalot F IT Study Group of the Italian Society of Diabetology Women show worse control of type 2 diabetes and cardiovascular disease risk factors than men: results from the MIND. Nutr Metab Cardiovasc Dis 2013;23:235-41. 10.1016/j.numecd.2011.12.003 22397873

[30] Winston GJ Barr RG Carrasquillo O Bertoni AG Shea S Sex and racial/ethnic differences in cardiovascular disease risk factor treatment and control among individuals with diabetes in the Multi-Ethnic Study of Atherosclerosis (MESA). Diabetes Care 2009;32:1467-9. 10.2337/dc09-0260 19435957 PMC2713610

