# Supplementary material for: Sex differences in risk factors for myocardial infarction: cohort study of UK Biobank participants
Source: BMJ. 2018 Nov 7;363:k4247. doi: 10.1136/bmj.k4247 (PMC6364292; doi:10.1136/bmj.k4247)
Supplement: Supplementary file 1 — Supplementary information: supplementary tables 1-9, and equations [file mile045856.ww1.pdf]

Supplementary table 1 Adjusted hazard ratios for incident myocardial infarction comparing women to men, by age

Age group (years)	Hazard ratio (95% CI)
<45	0.27 (0.18 to 0.41)
45-49	0.30 (0.23 to 0.38)
50-54	0.35 (0.29 to 0.42)
55-59	0.30 (0.26 to 0.36)
60-64	0.38 (0.34 to 0.43)
≥65	0.45 (0.40 to 0.50)

Adjusted for age, systolic blood pressure, body mass index, smoking status and diabetes

Supplementary table 2 Multiple adjusted hazard ratios and women-to-men ratios of hazard ratios for the association between American Heart Association (AHA) hypertension stages further classified according to self-reported anti-hypertensive use and incident myocardial infarction

Risk factor	Women (95% CI)	Men (95% CI)	RHR (95% CI)
Elevated blood pressure and no anti-hypertensive use	1.68 (1.27 to 2.22)	0.95 (0.76 to 1.20)	1.76 (1.23 to 2.53)
Stage 1 hypertension and no anti-hypertensive use	2.10 (1.67 to 2.65)	1.50 (1.24 to 1.80)	1.41 (1.05 to 1.89)
Stage 2 hypertension and no anti-hypertensive use	2.83 (2.23 to 3.61)	2.05 (1.70 to 2.46)	1.39 (1.02 to 1.88)
Normal BP and anti-hypertensive medication	2.81 (1.75 to 4.52)	2.49 (1.76 to 3.52)	1.13 (0.63 to 2.03)
Elevated BP and anti-hypertensive medication	3.65 (2.44 to 5.44)	1.75 (1.26 to 2.44)	2.08 (1.24 to 3.50)
Stage 1 hypertension and anti-hypertensive medication	3.14 (2.39 to 4.13)	1.85 (1.50 to 2.28)	1.70 (1.20 to 2.40)
Stage 2 hypertension and anti-hypertensive medication	4.24 (3.21 to 5.60)	2.22 (1.79 to 2.74)	1.91 (1.35 to 2.71)

Compared to participants with normal blood pressure at baseline, who did not report use of anti-hypertensive medication.

Adjusted for age, diabetes, socioeconomic status, smoking status, body mass index and use of lipid-lowering medication

Supplementary table 3 Multiple adjusted hazard ratios and women-to-men ratios of hazard ratios for the association between diabetes type II further classified according to self-reported treatment type, and incident myocardial infarction

Treatment, Type II diabetes	Women (95% CI)	Men (95% CI)	RHR (95% CI)
No medication reported	1.50 (1.07 to 2.10)	1.14 (0.93 to 1.39)	1.31 (0.88 to 1.95)
Oral medication only	1.93 (1.46 to 2.54)	1.33 (1.13 to 1.57)	1.45 (1.05 to 2.00)
Insulin only	4.02 (2.35 to 6.86)	2.20 (1.54 to 3.15)	1.82 (0.96 to 3.47)
Oral medication & insulin	3.64 (2.28 to 5.82)	1.69 (1.21 to 2.37)	2.15 (1.21 to 3.83)

Compared to participants without diabetes

Adjusted for age, systolic blood pressure, socioeconomic status, smoking status, body mass index, use of lipid-lowering medication and anti-hypertensive medication.

Supplementary table 4 Age adjusted hazard ratios for MI by risk factor in men and women, and women-to-men ratios of hazard ratios

Risk factor	Women (95% CI)	Men (95% CI)	RHR (95% CI)
SBP/20 unit increase	1.40 (1.33 to 1.47)	1.31 (1.26 to 1.36)	1.07 (1.01 to 1.14)
DBP/10 unit increase	1.26 (1.19 to 1.32)	1.24 (1.20 to 1.28)	1.02 (0.96 to 1.08)
AHA Hypertension stages			
Elevated blood pressure	1.67 (1.31 to 2.14)	0.90 (0.74 to 1.10)	1.85 (1.35 to 2.53)
Stage 1 hypertension	1.98 (1.61 to 2.42)	1.38 (1.18 to 1.62)	1.43 (1.11 to 1.85)
Stage 2 hypertension	2.71 (2.20 to 3.35)	1.87 (1.60 to 2.19)	1.45 (1.12 to 1.89)
Smoking status			
Ex-smoker	1.28 (1.14 to 1.44)	1.21 (1.12 to 1.30)	1.06 (0.92 to 1.22)
Current smoker	3.74 (3.27 to 4.28)	2.31 (2.11 to 2.52)	1.62 (1.38 to 1.90)
Current smoking, by cigarette consumption			
1-9/day	2.24 (1.60 to 3.15)	1.84 (1.41 to 2.41)	1.22 (0.79 to 1.88)
10-19/day	3.94 (3.23 to 4.80)	2.79 (2.41 to 3.22)	1.41 (1.11 to 1.80)
20+/day	6.19 (5.08 to 7.55)	3.11 (2.72 to 3.54)	1.99 (1.57 to 2.53)
Diabetes			
Type I	10.90 (7.08 to 16.78)	2.80 (1.82 to 4.30)	3.89 (2.12 to 7.15)
Type II	2.65 (2.22 to 3.16)	1.55 (1.39 to 1.73)	1.71 (1.39 to 2.11)
BMI			
BMI/5 unit increase	1.22 (1.17 to 1.28)	1.25 (1.21 to 1.30)	0.98 (0.92 to 1.04)
Overweight	1.17 (1.03 to 1.33)	1.29 (1.18 to 1.41)	0.91 (0.78 to 1.06)
Obese	1.63 (1.43 to 1.85)	1.70 (1.55 to 1.87)	0.95 (0.81 to 1.12)
History of atrial fibrillation	1.62 (0.94 to 2.80)	1.29 (0.99 to 1.67)	1.26 (0.69 to 2.31)
SES third (Townsend)			
Middle	1.24 (1.10 to 1.40)	1.07 (0.99 to 1.15)	1.17 (1.01 to 1.35)
Lowest	1.69 (1.48 to 1.92)	1.32 (1.21 to 1.44)	1.28 (1.10 to 1.49)

Systolic blood pressure (SBP) per 20 mmMg, diastolic blood pressure (DBP) per 10 mmMg, current smokers were compared with never smokers, participants with diabetes were compared with those without diabetes, obese and overweight participants were compared with those with BMI<25kg/m^2, middle and lowest thirds of socioeconomic status (SES) were compared with highest third.

Supplementary table 5 Multiple adjusted hazard ratios for the association between risk factors and incident myocardial infarction, by age group and sex

Risk factor	Women	p-trend	Men	p-trend	RHR (W:M)	p-interaction
Systolic BP		0.001		<0.001		0.66
<50 years	1.69 (1.39 to 2.05)		1.62 (1.44 to 1.83)		1.04 (0.83 to 1.30)	
50-59 years	1.57 (1.43 to 1.73)		1.32 (1.23 to 1.41)		1.19 (1.06 to 1.34)	
≥60 years	1.34 (1.26 to 1.43)		1.25 (1.20 to 1.31)		1.07 (0.99 to 1.16)	
Diastolic BP		0.03		<0.001		0.24
<50 years	1.44 (1.22 to 1.70)		1.54 (1.40 to 1.68)		0.93 (0.77 to 1.13)	
50-59 years	1.27 (1.15 to 1.40)		1.26 (1.18 to 1.33)		1.01 (0.90 to 1.13)	
≥60 years	1.19 (1.12 to 1.27)		1.13 (1.08 to 1.18)		1.05 (0.97 to 1.14)	
Elevated BP		0.10		0.29		0.51
<50 years	2.31 (1.21 to 4.41)		1.11 (0.64 to 1.93)		2.08 (0.89 to 4.86)	
50-59 years	1.86 (1.19 to 2.90)		0.88 (0.63 to 1.23)		2.11 (1.21 to 3.68)	
≥60 years	1.32 (0.94 to 1.85)		0.80 (0.61 to 1.06)		1.65 (1.06 to 2.55)	
Stage 1 hypertension		0.08		0.04		0.91
<50 years	2.36 (1.40 to 3.99)		1.92 (1.23 to 2.99)		1.23 (0.62 to 2.45)	
50-59 years	2.35 (1.63 to 3.38)		1.30 (1.00 to 1.69)		1.80 (1.15 to 2.82)	
≥60 years	1.60 (1.21 to 2.13)		1.17 (0.94 to 1.46)		1.37 (0.96 to 1.97)	
Stage 2 hypertension		0.03		0.001		0.78
<50 years	3.53 (1.96 to 6.36)		3.77 (2.40 to 5.90)		0.94 (0.45 to 1.97)	
50-59 years	3.29 (2.25 to 4.81)		1.69 (1.29 to 2.20)		1.95 (1.23 to 3.10)	
≥60 years	2.07 (1.55 to 2.76)		1.49 (1.19 to 1.86)		1.39 (0.97 to 2.00)	
Ex-smokers		0.39		0.82		0.54
<50 years	1.37 (0.86 to 2.18)		1.11 (0.85 to 1.45)		1.23 (0.72 to 2.11)	
50-59 years	1.36 (1.07 to 1.72)		1.26 (1.10 to 1.45)		1.07 (0.81 to 1.41)	
≥60 years	1.21 (1.05 to 1.40)		1.21 (1.10 to 1.32)		1.01 (0.85 to 1.19)	
Current smokers		0.20		<0.001		0.49
<50 years	3.63 (2.36 to 5.57)		2.63 (2.09 to 3.30)		1.38 (0.85 to 2.25)	
50-59 years	4.05 (3.17 to 5.16)		2.61 (2.24 to 3.04)		1.55 (1.16 to 2.06)	
≥60 years	3.11 (2.59 to 3.73)		1.80 (1.58 to 2.05)		1.73 (1.38 to 2.16)	
Smoke 1-9/day		0.88		0.75		0.76
<50 years	0.56 (0.08 to 4.05)		1.33 (0.65 to 2.70)		0.42 (0.05 to 3.45)	
50-59 years	3.33 (1.96 to 5.65)		2.50 (1.67 to 3.74)		1.33 (0.68 to 2.59)	
≥60 years	1.94 (1.23 to 3.08)		1.45 (0.96 to 2.19)		1.34 (0.72 to 2.49)	
Smoke 10-19/day		0.46		0.04		0.62
<50 years	4.38 (2.42 to 7.95)		3.31 (2.37 to 4.63)		1.32 (0.67 to 2.62)	
50-59 years	3.99 (2.79 to 5.71)		2.93 (2.30 to 3.74)		1.36 (0.88 to 2.10)	
≥60 years	3.54 (2.72 to 4.61)		2.22 (1.79 to 2.77)		1.59 (1.13 to 2.24)	
Smoke ≥20/day		0.02		<0.001		0.94
<50 years	8.64 (5.02 to 14.85)		4.36 (3.25 to 5.85)		1.98 (1.07 to 3.67)	
50-59 years	6.64 (4.75 to 9.27)		3.13 (2.52 to 3.90)		2.12 (1.42 to 3.16)	
≥60 years	4.61 (3.46 to 6.15)		2.16 (1.75 to 2.68)		2.13 (1.49 to 3.05)	
Type I diabetes		0.71		0.18		0.22
<50 years	7.77 (2.45 to 24.58)		3.18 (1.18 to 8.53)		2.44 (0.54 to 11.14)	
50-59 years	8.43 (4.16 to 17.10)		0.69 (0.17 to 2.77)		12.20 (2.57 to 58.00)	
≥60 years	6.45 (3.31 to 12.55)		4.08 (2.45 to 6.82)		1.58 (0.68 to 3.66)	

Type II diabetes	0.15	0.05	0.83
<50 years	3.73 (1.93 to 7.20)	2.18 (1.43 to 3.31)	1.71 (0.79 to 3.73)
50-59 years	1.96 (1.33 to 2.89)	1.32 (1.05 to 1.66)	1.49 (0.95 to 2.34)
≥60 years	1.90 (1.51 to 2.41)	1.30 (1.12 to 1.50)	1.47 (1.11 to 1.93)
BMI (/5 units)	0.12	0.13	0.75
<50 years	1.32 (1.16 to 1.51)	1.32 (1.19 to 1.45)	1.00 (0.85 to 1.18)
50-59 years	1.24 (1.14 to 1.35)	1.28 (1.20 to 1.36)	0.97 (0.88 to 1.08)
≥60 years	1.18 (1.12 to 1.26)	1.22 (1.16 to 1.28)	0.97 (0.90 to 1.05)
Overweight	0.74	0.46	0.91
<50 years	1.37 (0.87 to 2.16)	1.68 (1.27 to 2.22)	0.81 (0.48 to 1.39)
50-59 years	1.14 (0.89 to 1.46)	1.22 (1.04 to 1.43)	0.94 (0.70 to 1.25)
≥60 years	1.19 (1.02 to 1.39)	1.34 (1.20 to 1.50)	0.88 (0.73 to 1.07)
Obese	0.13	0.04	0.92
<50 years	2.12 (1.35 to 3.33)	2.38 (1.77 to 3.19)	0.89 (0.52 to 1.53)
50-59 years	1.71 (1.34 to 2.18)	1.66 (1.40 to 1.96)	1.03 (0.77 to 1.39)
≥60 years	1.51 (1.28 to 1.79)	1.61 (1.42 to 1.83)	0.94 (0.76 to 1.16)
History of AF	0.13	0.31	0.38
<50 years	8.14 (1.13 to 58.48)	2.11 (0.53 to 8.48)	3.85 (0.35 to 43.02)
50-59 years	2.25 (0.56 to 9.04)	1.64 (0.90 to 2.97)	1.37 (0.30 to 6.23)
≥60 years	1.41 (0.75 to 2.63)	1.32 (0.98 to 1.79)	1.06 (0.53 to 2.13)
SES mid vs high	0.52	0.76	0.48
<50 years	1.31 (0.83 to 2.08)	0.91 (0.70 to 1.17)	1.44 (0.85 to 2.45)
50-59 years	1.18 (0.93 to 1.50)	1.05 (0.91 to 1.21)	1.12 (0.85 to 1.48)
≥60 years	1.12 (0.96 to 1.30)	0.99 (0.90 to 1.10)	1.13 (0.94 to 1.35)
SES low vs high	0.12	0.06	0.70
<50 years	1.59 (1.01 to 2.51)	1.36 (1.08 to 1.73)	1.17 (0.70 to 1.95)
50-59 years	1.44 (1.12 to 1.84)	1.18 (1.01 to 1.38)	1.22 (0.91 to 1.63)
≥60 years	1.16 (0.98 to 1.38)	1.04 (0.92 to 1.17)	1.12 (0.91 to 1.38)

Systolic blood pressure (SBP) per 20 mmMg, diastolic blood pressure (DBP) per 10 mmMg. Hypertension stages were compared to participants with normal blood pressure, smoking groups were compared with never smokers, participants with diabetes were compared with those without diabetes, overweight and obese were compared with BMI <25kg/m^2, patients with Atrial Fibrillation (AF) were compared to those without AF.

All models were adjusted for age. Additionally, SBP, diabetes and SES were adjusted for each other as well as smoking status, BMI, use of lipid-lowering medication and anti-hypertensive medication. AF was similarly adjusted for these eight variables. DBP and AHA hypertension stages were adjusted for the same variables as SBP. The models for smoking variables included SES, and the models for BMI contained smoking status and SES.

Supplementary table 6 Multiple adjusted hazard ratios for the association between risk factors and incident myocardial infarction, by socioeconomic status and sex

	Women	Men	RHR	p-interaction
Systolic BP				0.94
Higher SES	1.38 (1.29 - 1.47)	1.27 (1.21 - 1.33)	1.09 (1.00 - 1.18)	
Lower SES	1.40 (1.30 - 1.52)	1.30 (1.22 - 1.38)	1.08 (0.98 - 1.20)	
Diastolic BP				0.96
Higher SES	1.22 (1.14 - 1.30)	1.20 (1.15 - 1.25)	1.01 (0.94 - 1.10)	
Lower SES	1.27 (1.17 - 1.37)	1.25 (1.19 - 1.32)	1.01 (0.92 - 1.11)	
Elevated BP				0.99
Higher SES	1.75 (1.25 - 2.45)	0.95 (0.74 - 1.22)	1.84 (1.21 - 2.81)	
Lower SES	1.28 (0.88 - 1.87)	0.69 (0.50 - 0.97)	1.85 (1.12 - 3.06)	
Stage 1 hypertension				0.31
Higher SES	2.19 (1.65 - 2.92)	1.32 (1.08 - 1.62)	1.66 (1.17 - 2.36)	
Lower SES	1.53 (1.13 - 2.07)	1.21 (0.94 - 1.55)	1.27 (0.86 - 1.87)	
Stage 2 hypertension				0.76
Higher SES	2.62 (1.94 - 3.53)	1.77 (1.43 - 2.18)	1.48 (1.03 - 2.13)	
Lower SES	2.30 (1.68 - 3.14)	1.68 (1.30 - 2.16)	1.37 (0.91 - 2.04)	
Ex-smokers				0.004
Higher SES	1.14 (0.98 - 1.32)	1.24 (1.14 - 1.36)	0.91 (0.77 - 1.09)	
Lower SES	1.49 (1.23 - 1.82)	1.06 (0.93 - 1.22)	1.41 (1.11 - 1.79)	
Current smokers				0.20
Higher SES	3.11 (2.56 - 3.79)	2.19 (1.94 - 2.47)	1.42 (1.13 - 1.79)	
Lower SES	4.07 (3.33 - 4.98)	2.30 (2.00 - 2.64)	1.77 (1.39 - 2.26)	
Smoke 1-9/day				0.21
Higher SES	2.32 (1.48 - 3.63)	1.42 (0.93 - 2.16)	1.64 (0.89 - 3.03)	
Lower SES	2.08 (1.23 - 3.52)	2.22 (1.57 - 3.15)	0.94 (0.50 - 1.76)	
Smoke 10-19/day				0.23
Higher SES	3.53 (2.63 - 4.74)	2.96 (2.42 - 3.62)	1.19 (0.83 - 1.70)	
Lower SES	4.06 (3.07 - 5.36)	2.51 (2.03 - 3.10)	1.62 (1.14 - 2.29)	
Smoke ≥20/day				0.27
Higher SES	4.87 (3.50 - 6.78)	2.94 (2.40 - 3.61)	1.65 (1.12 - 2.44)	
Lower SES	6.64 (5.11 - 8.63)	3.03 (2.52 - 3.63)	2.19 (1.59 - 3.02)	
Type I diabetes				0.94
Higher SES	7.96 (4.22 – 15.00)	2.95 (1.70 - 5.11)	2.70 (1.17 - 6.24)	
Lower SES	7.63 (4.04 - 14.38)	2.69 (1.34 - 5.42)	2.83 (1.10 - 7.27)	
Type II diabetes				0.79
Higher SES	2.04 (1.55 - 2.67)	1.40 (1.20 - 1.63)	1.46 (1.07 - 1.99)	
Lower SES	1.96 (1.49 - 2.58)	1.27 (1.06 - 1.53)	1.54 (1.11 - 2.15)	
BMI (/5 units)				0.59
Higher SES	1.25 (1.17 - 1.33)	1.27 (1.21 - 1.33)	0.98 (0.91 - 1.06)	
Lower SES	1.19 (1.11 - 1.27)	1.25 (1.18 - 1.32)	0.95 (0.87 - 1.04)	
Overweight				0.74
Higher SES	1.14 (0.97 - 1.34)	1.28 (1.14 - 1.42)	0.90 (0.74 - 1.09)	
Lower SES	1.23 (1.00 - 1.51)	1.45 (1.25 - 1.69)	0.85 (0.66 - 1.10)	
Obese				0.40
Higher SES	1.68 (1.42 - 1.99)	1.69 (1.50 - 1.90)	0.99 (0.81 - 1.22)	
Lower SES	1.53 (1.24 - 1.89)	1.78 (1.52 - 2.09)	0.86 (0.66 - 1.12)	

History of AF				0.48
Higher SES	1.73 (0.90 - 3.35)	1.30 (0.94 - 1.79)	1.34 (0.64 - 2.78)	
Lower SES	1.23 (0.46 - 3.28)	1.47 (0.92 - 2.34)	0.83 (0.28 - 2.48)	

Hypertension stages were compared to participants with normal blood pressure, smoking groups were compared with never smokers, participants with diabetes were compared with those without diabetes, overweight and obese were compared with BMI <25kg/m^2, patients with Atrial Fibrillation (AF) were compared to those without a AF.

All models were adjusted for age. Additionally, SBP and diabetes were adjusted for each other as well as smoking status, BMI, use of lipid-lowering medication and anti-hypertensive medication. AF was similarly adjusted for these seven variables. DBP and AHA hypertension stages were adjusted for the same variables as SBP. The models for smoking variables were only adjusted for age, and the models for BMI also contained smoking status.

Supplementary table 7 Unadjusted rates of myocardial infarction (per 10,000 person-years) by sex, and difference of rate differences (women – men) for each risk factor

	Women	Men	Difference of rate difference
AHA Hypertension categories			
Normal	2.88 (2.34 to 3.41)	14.02 (11.93 to 16.11)	
Elevated	6.22 (5.23 to 7.20)	13.54 (11.77 to 15.32)	3.81 (0.85 to 6.78)
Stage 1 hypertension	8.07 (7.46 to 8.68)	22.95 (21.85 to 24.04)	-3.74 (-6.23 to -1.24)
Stage 2 hypertension	12.40 (11.34 to 13.47)	33.17 (31.50 to 34.84)	-9.63 (-12.55 to -6.70)
Smoking Status			
Never smoker	5.83 (5.38 to 6.27)	18.54 (17.57 to 19.52)	
Ex-smoker	8.17 (7.43 to 8.90)	26.55 (25.18 to 27.91)	-5.66 (-7.55 to -3.78)
Current smoker	18.78 (16.70 to 20.87)	40.84 (37.91 to 43.76)	-9.34 (-13.09 to -5.59)
Current smokers, by cigarette consumption			
Never	5.83 (5.38 to 6.27)	18.54 (17.57 to 19.52)	
1-9 per day	11.53 (7.71 to 15.35)	30.92 (22.82 to 39.02)	-6.67 (-15.69 to 2.35)
10-19	20.25 (16.56 to 23.93)	47.89 (41.46 to 54.32)	-14.93 (-22.41 to -7.44)
≥20	30.95 (25.34 to 36.56)	52.96 (46.57 to 59.35)	-9.29 (-17.86 to -0.72)
Diabetes diagnosis			
No diabetes	7.12 (6.73 to 7.51)	23.06 (22.26 to 23.86)	
Type I*	59.47 (34.03 to 84.90)	56.51 (32.34 to 80.68)	18.90 (-16.20 to 53.99)
Type II	22.60 (18.83 to 26.37)	42.50 (38.08 to 46.93)	-3.96 (-9.85 to 1.92)
Body Mass Index			
Normal (BMI <25)	5.97 (5.42 to 6.52)	18.11 (16.77 to 19.46)	
Overweight (BMI ≥25, <30)	7.84 (7.18 to 8.50)	24.06 (22.94 to 25.19)	-4.08 (-6.04 to -2.13)
Obese (BMI ≥30)	10.51 (9.54 to 11.48)	31.23 (29.40 to 33.06)	-8.57 (-11.10 to -6.04)
Atrial fibrillation			
None	7.72 (7.32 to 8.11)	24.20 (23.40 to 24.99)	
History of atrial fibrillation	18.58 (8.48 to 28.67)	40.31 (29.85 to 50.78)	-5.26 (-19.83 to 9.31)
Townsend in thirds			
High	6.76 (6.24 to 7.28)	23.49 (22.40 to 24.58)	
Middle	7.99 (7.25 to 8.72)	23.74 (22.28 to 25.20)	0.98 (-1.05 to 3.00)
Low	10.17 (9.11 to 11.22)	27.64 (25.73 to 29.55)	-0.75 (-3.24 to 1.75)

Supplementary table 8 Number of women and men with missing information at baseline

	Women n(%)	Men n(%)
Age	0 (0.0)	0 (0.0)
Ethnicity	443 (0.2)	420 (0.2)
Systolic blood pressure	724 (0.3)	508 (0.2)
Diastolic blood pressure	722 (0.3)	508 (0.2)
Hypertension	724 (0.3)	508 (0.2)
Hypertension including medication use	724 (0.3)	508 (0.2)
Smoking status	1429 (0.5)	1257 (0.5)
Body mass index	1347 (0.5)	1405 (0.7)
Socioeconomic status	317 (0.1)	273 (0.1)
Diabetes	1084 (0.4)	1174 (0.6)
Cigarettes per day (among current smokers)	5689 (24.6)	9982 (38.7)

Missing data includes non-response and responses “don’t know” and “prefer not to answer”

Supplementary table 9 Comparison of complete case and multiply imputed analyses of daily cigarette consumption level among current smokers

		Women	Men	RHRs
Complete case	Current smoking, 10-19/day	1.71 (1.17 to 2.49)	1.52 (1.13 to 2.03)	1.13 (0.70 to 1.82)
	Current smoking, 20+/day	2.62 (1.79 to 3.83)	1.67 (1.25 to 2.23)	1.57 (0.97 to 2.53)
Multiply imputed	Current smoking, 10-19/day	1.64 (1.14 to 2.36)	1.40 (1.04 to 1.87)	1.17 (0.74 to 1.86)
	Current smoking, 20+/day	2.45 (1.69 to 3.56)	1.64 (1.21 to 2.22)	1.49 (0.93 to 2.40)

Compared to smokers of 1-9 cigarettes per day. Adjusted for age and socioeconomic status.

Supplementary text

Let P be the prevalence of a cluster of adverse risk factors, and assume this represents the full effect of risk factors. Assume P is the same in women and men. By definition, $0 \le P \le 1$.

Let w be the risk of MI in unexposed (to the cluster) women.

Let s be the male:female risk ratio for MI in unexposed women.

Let r be the exposed:unexposed relative risk for MI in women.

Let t be the male:female ratio of relative risks for MI.

Then the risk of MI in women overall is

$$Prw + (1 - P)w$$

and the risk in men is

$$Ptrsw + (1 - P)sw.$$

So for the female risk to equal the male risk,

$$Prw + (1 - P)w = Ptrsw + (1 - P)\,sw,$$

$$\text{i.e. } Pr + (1 - P) = Ptrs + (1 - P)s.$$

If we consider all else to be fixed, this equates to

$$P = \frac{s-1}{r+s-trs-1} = E.$$

For example, if $r = 3$ (women with the risk cluster are 3 times as likely to get MI as are those without), $s = 2$ (men without the risk cluster are twice as likely to get MI as are their female peers) and $t = 1/3$ (the relative risk for women is three times that of men), women and men will have equal rates of MI if $P = 0.5$ (that is, if 50% of people have all the risk factors in the cluster).

If $E > 0$ then the overall female rate will exceed the male rate if $P > E$,

if $E < 0$ then the male rate will always exceed the female rate.

So, in the example, women will have higher rates of MI if the (percentage) prevalence of the risk cluster exceeds 50%. In reality, this is extremely unlikely.
